# Supplementary material for: Dually Responsive Nanoparticles for Drug Delivery Based on Quaternized Chitosan
Source: Int J Mol Sci. 2022 Jul 1;23(13):7342. doi: 10.3390/ijms23137342 (PMC9266538; doi:10.3390/ijms23137342)
Supplement: Supplementary file 1 [file ijms-23-07342-s001.zip › ijms-1742737-supplementary.pdf]

Supplementary Information for

Dually responsive nanoparticles for drug delivery based on quaternized chitosan

Fenghui Qiao[1], Zhiqi Jiang[1] , Wen Fang[1], Jing Zhi Sun[1,2] and Qiaoling Hu[1,*]

1 MOE Key Laboratory of Macromolecular Synthesis and Functionalization, Department of Polymer Science and Engineering, Zhejiang University, Hangzhou 310027, China; huql@zju.edu.cn

2 Center of Healthcare Materials, Shaoxing Institute, Zhejiang University, Shaoxing, 312000, China; sunjz@zju.edu.cn

*Correspondence: huql@zju.edu.cn

I. Supplementary Tables

Table S1 Integration information from the ^{1}H NMR spectrum of PQCS.

Protons of different units	δ (ppm)	Integration	Relative amount of units
-C<u>H</u>NH$_2$	2.7-3.0	0.33	0.33/1
-N$^+$(C<u>H</u>$_3$)$_3$	3.2	1.37	1.37/9
Ar	7.7-7.8	0.28	0.28/4

Setting the total repeating sugar ring units in chitosan as 100%; degree of substitution of quaternary ammonium units (D.S. q) values and degree of substitution of phenylboronic acid units (D.S. p) values were calculated based on the data shown in Table S1 using the following equations:

D.S. q = [(1.37/9)/(0.33/1)]*100% = 46.1%

D.S. p = [(0.28/4)/(0.33/1)]*100% = 21.2%

D.S. = degree of substitution.

Table S2 DOX loading content and DOX loading efficiency of Hep/PQCS-PVA nanoparticles.

Sample	DLC[a]	DLE[b]
DOX (10 μg mL^{-1})-loaded Hep/PQCS-PVA	2.0%	99.5%
DOX (25 μg mL^{-1})-loaded Hep/PQCS-PVA	5.0%	99.4%
DOX (50 μg mL^{-1})-loaded Hep/PQCS-PVA	9.4%	98.9%

[a] DOX loading content (DLC) and [b] DOX loading efficiency (DLE) were calculated using the following equations:

$$DLC = \left[\frac{\text{weight of loaded DOX}}{\text{weight of DOX} - \text{loaded nanoparticle}} \right] \times 100\%$$

$$DLE = \left[\frac{\text{weight of loaded DOX}}{\text{weight of DOX in feeding}} \right] \times 100\%$$

Table S3 IC_{50} of DOX-loaded Hep/PQCS-PVA nanoparticles and DOX after incubating for 1 day.

Cell type	IC_{50} of DOX-loaded Hep/PQCS-PVA nanoparticles	IC_{50} of DOX-loaded Hep/PQCS-PVA nanoparticles	IC_{50} of DOX
Hep G2	44.3 μg mL^{-1}	4.18 μg DOX equiv. mL^{-1}	2.24 μg mL^{-1}
HeLa	3220.60 μg mL^{-1}	303.83 μg DOX equiv. mL^{-1}	65.53 μg mL^{-1}

II. Supplementary Scheme and Figures

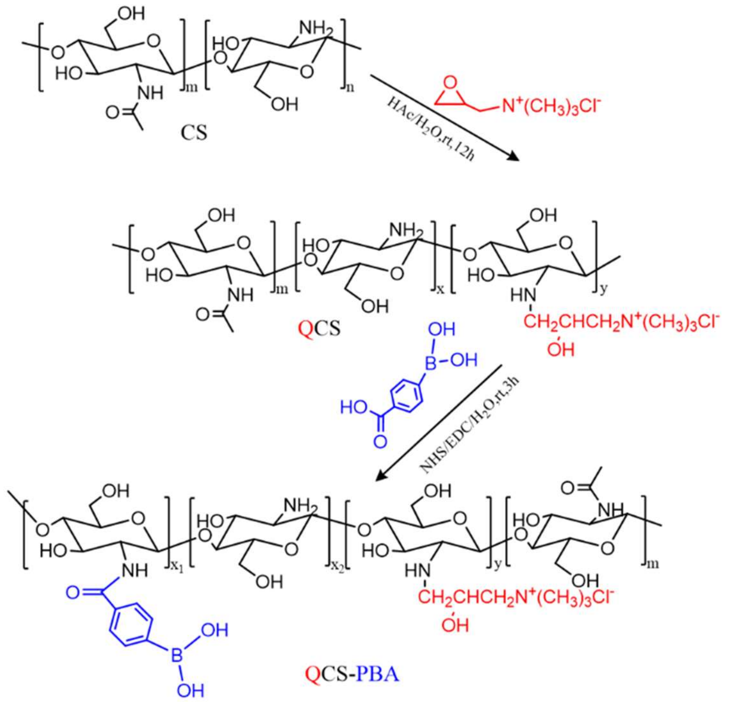

Scheme S1 Synthetic route to PQCS.

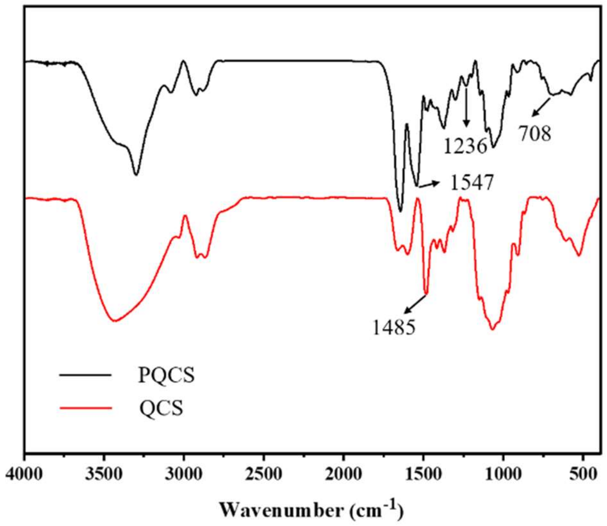

Figure S1 FTIR spectra of QCS and PQCS. The samples were prepared via potassium bromide pellet technique.

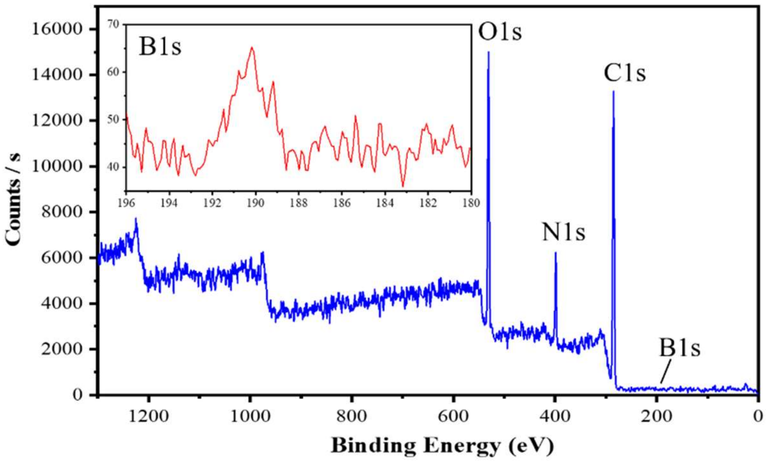

Figure S2 XPS survey spectrum of PQCS and high resolution XPS spectrum (inset) of the B1s region for corresponding sample.

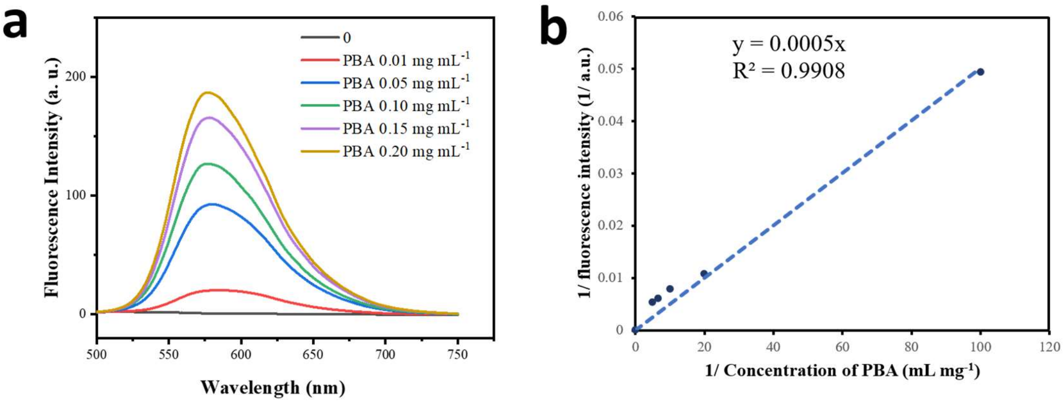

Figure S3 (a) Fluorescence spectra of AR (0.034 mg mL^{-1})/PBA PBS solutions, excitation: 468 nm; (b) Standard curve between 1/I and 1/C. I = fluorescence intensity and C = concentration of PBA calculated from (a).

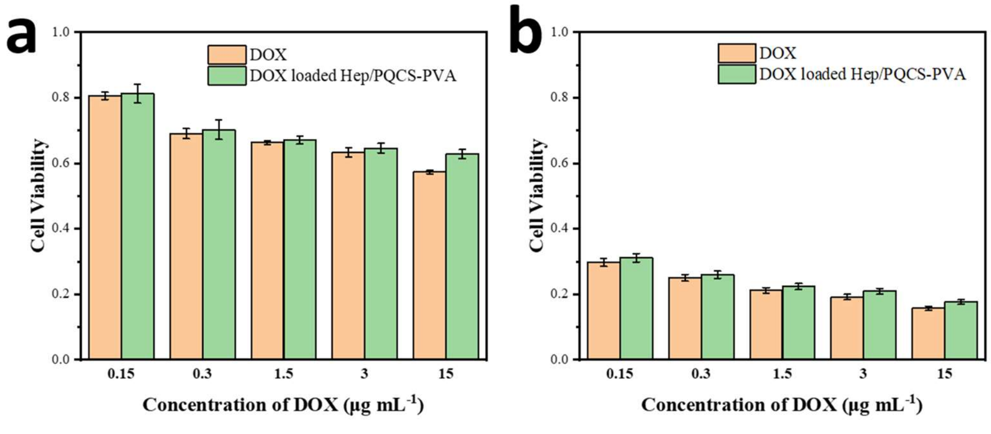

Figure S4 Cell viability of DOX-loaded Hep/PQCS-PVA nanoparticles and free DOX toward

HeLa cells at (a) 1st day and (b) 3rd day.

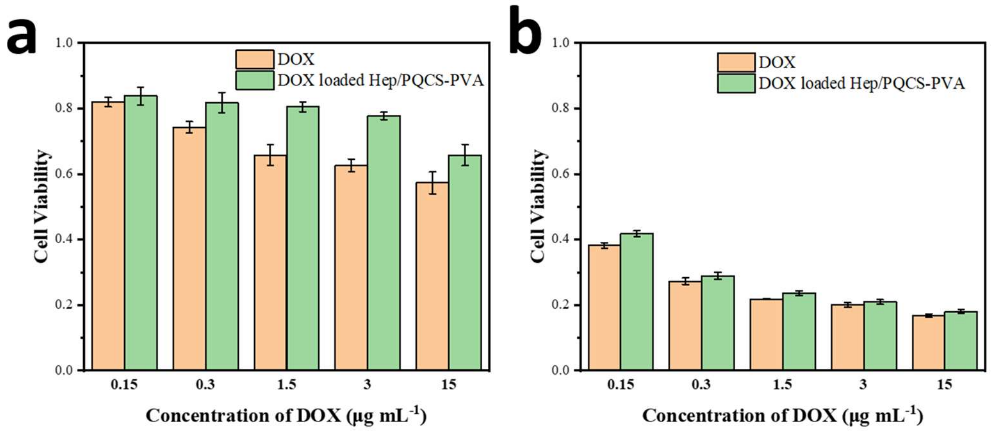

Figure S5 Cell viability of DOX-loaded Hep/PQCS-PVA nanoparticles and free DOX toward FIB cells at (a) 1st day and (b) 3rd day.

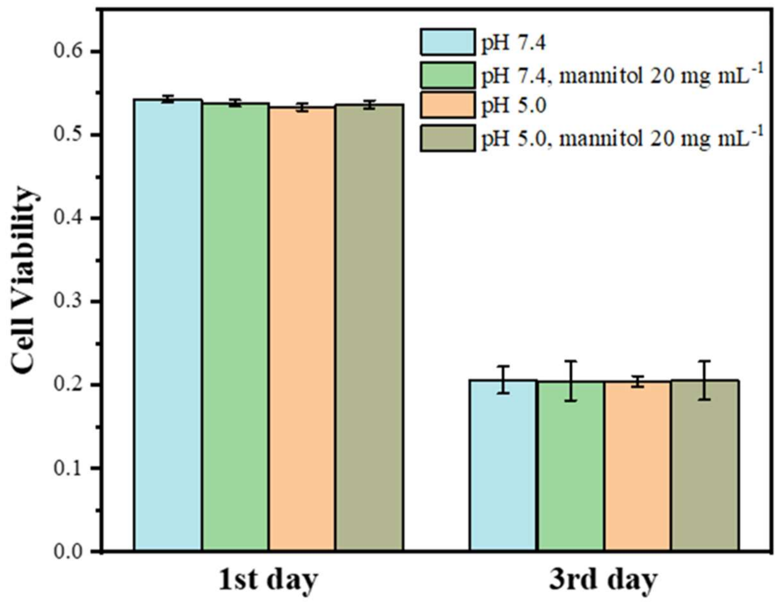

Figure S6 Cell viability of DOX toward Hep G2 cells with or without treatment with 20 mg mL^{-1} mannitol at pH 7.4 or pH 5.0.

III. Supplementary Discussion

The FTIR spectra (Supplementary Figure S1) of QCS and PQCS revealed the expected structure as designed. The peak at 1485 cm^{-1} in QCS spectrum belonged to the characteristic peak of the amine NH vibration deformation, whereas it disappeared in PQCS spectrum that suggested the increasing of substitution of the amino groups in PQCS. Moreover, the peaks at 1547 cm^{-1} and 708 cm^{-1} arising in PQCS spectrum were assigned to C-C stretches and C-H out-of-plane bending bonds in the aromatic ring of PBA units, respectively. There was also a characteristic absorption peak of $-B(OH)_2$ groups appearing at 1236 cm^{-1} in PQCS spectrum, which further suggested the successful substitution of PBA on QCS backbones.[1-3]

In order to analyze chemical composition of the prepared PQCS, the samples were characterized by XPS. As shown in Supplementary Figure S2, the sample contained O, N, C and B elements, with sharp photoelectron peaks appearing around binding energies of 528 eV (O 1s), 401 eV (N 1s) and 285 eV (C 1s) and a weak photoelectron peak around 190 eV (B 1s). The boron elements in PQCS were ascribed to phenylboronic acid groups grafted on QCS.

A calibration curve was prepared by plotting the fluorescence intensity at 579 nm as function of concentrations of 4-carboxyphenylboronic acid (Supplementary Figure S3b). Based on the calibration curve, the degree of substitution of PBA on the PQCS was calculated from the fluorescence intensity of samples (0.2 mg mL^{-1}).

References

1. R. Smoum, A. Rubinstein and M. Srebnik, Chitosan-pentaglycine-phenylboronic acid conjugate: A potential colon-specific platform for calcitonin, *Bioconjugate Chemistry*, 2006, **17**, 1000-1007.
2. D. Zhang, G. Yu, Z. Long, G. Yang and B. Wang, Controllable layer-by-layer assembly of PVA and phenylboronic acid-derivatized chitosan, *Carbohydr Polym*, 2016, **140**, 228-232.
3. L. Zhao, Y. Zhang, J. Shao, H. Liang, H. Na and J. Zhu, Folate-conjugated dually responsive micelles for targeted anticancer drug delivery, *RSC Adv.*, 2016, **6**, 35658-35667.
